# An atypical case of lymphoproliferative pulmonary involvement in a patient with Sjögren’s syndrome: a case report

**DOI:** 10.1186/1756-0500-6-367

**Published:** 2013-09-11

**Authors:** Hiroaki Oka, Hiroshi Ishii, Kosaku Komiya, Hisako Kushima, Chie Yasuda, Jun-ichi Kadota

**Affiliations:** 1 Department of Respiratory Medicine and Infectious Diseases, Oita University Faculty of Medicine, Oita, Japan; 2 Department of Respiratory Medicine, Faculty of Medicine, Fukuoka University, 7-45-1 Nanakuma, Jonan-ku, Fukuoka 814-0180, Japan

**Keywords:** Sjögren’s syndrome, Lymphoproliferative pulmonary disease, T-cells

## Abstract

**Background:**

Sjögren’s syndrome is characterized by lymphocytic infiltration of the exocrine glands, together with polyclonal B-cell activation, and lung diseases are well-known complications of the disease. Therefore, in most cases associated with Sjögren’s syndrome, infiltrating lymphocytes in the lung specimen exhibit the features of B-cells. We herein report an atypical case of lymphoproliferative pulmonary involvement in a patient with Sjögren’s syndrome.

**Case presentation:**

A 46-year-old female was admitted to our hospital because of an abnormal chest roentgenogram finding on a medical checkup. Chest computed tomography showed randomly-distributed micronodules and patchy ground-glass opacities. A surgical biopsied specimen showed an atypical pattern of interstitial pneumonia with numerous lymphoid follicles. Among the infiltrating lymphocytes in the lung, only the monoclonality of the T-cells was proven by a gene rearrangement analysis, but there was no cytological atypicality or genetic disorder revealed by testing the bone marrow aspirate. A diagnosis of Sjögren’s syndrome was made based on the patient’s other symptoms and these negative findings. The patient’s pulmonary lesions have been successfully treated and remission has been maintained for over three years with corticosteroid treatment alone.

**Conclusion:**

The present patient was an atypical case of lymphoproliferative pulmonary involvement in a patient with Sjögren’s syndrome. Although monoclonality of the infiltrating T-cells was proven, the clinical course and the findings of the imaging and laboratory examinations were inconsistent with the previously-reported cases of primary pulmonary T-cell lymphoma. This suggests that the monoclonality of lymphocytes does not always define malignancy. The diagnosis of malignant lymphoma or lymphoproliferative diseases should be made clinically, pathologically and cytogenetically to rule out other similar diseases.

## Background

Sjögren’s syndrome (SS) is characterized by lymphocytic infiltrates in the exocrine glands, together with polyclonal B-cell activation [1]. Lung diseases are well-known complications of SS, which comprises various lymphoproliferative disorders. Interstitial lung disease due to SS commonly presents as nonspecific interstitial pneumonia or lymphoid interstitial pneumonia (LIP) [2,3], and the term has been widely used to describe supposedly benign or reactive lymphoid hyperplasia. We herein report an atypical case of lymphoproliferative pulmonary involvement in a patient with Sjögren’s syndrome.

## Case presentation

**Figure 1:**
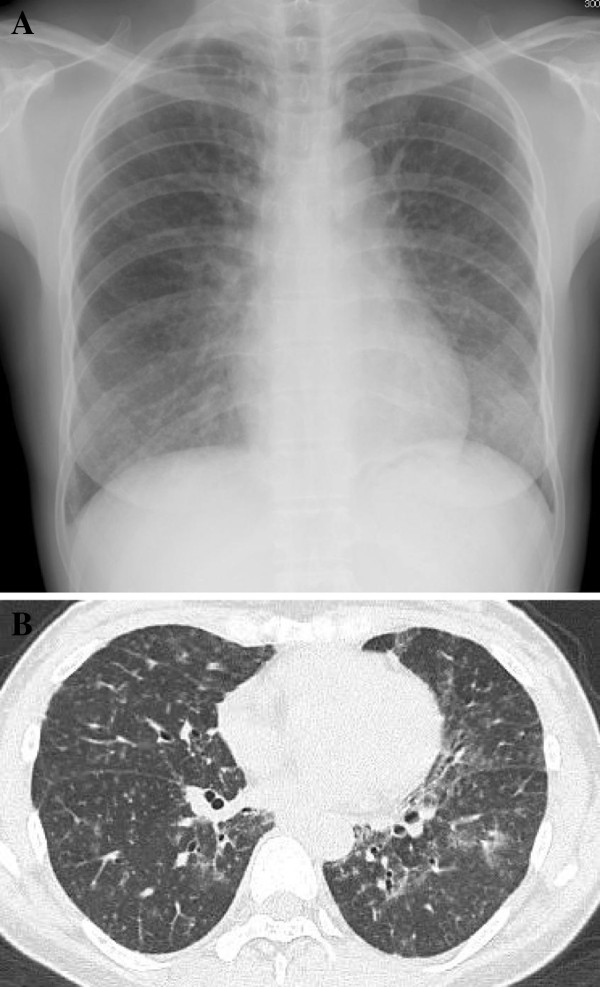
**Radiological findings.** A chest X-ray on admission, showing fine and diffuse nodular shadows in both lung fields **(A)**. An initial CT scan of the chest demonstrated randomly-distributed micronodules and patchy ground-glass opacities **(B)**.

A 46-year-old female was admitted to our hospital after the identification of abnormal chest X-ray shadows during a medical checkup (Figure 1A). She had no history of either smoking or dust inhalation, behaviors associated with a risk of human immunodeficiency virus (HIV) infection or any other appreciable disease. She had a family history of rheumatoid arthritis in both her sister and grandmother. She complained of slightly dry eyes, but had no fever, arthralgia, myalgia or dryness of the mouth. Her vital signs were within the normal ranges. A physical examination disclosed no finger clubbing, crackles on chest auscultation or swollen superficial lymph nodes. The laboratory tests revealed a normal blood count and routine chemistry findings. The serum levels of KL-6 and surfactant protein-D, markers for interstitial pneumonia, were 946 (normal value <500) U/mL and 178 (<110) ng/mL, respectively. An examination for autoantibodies showed a positive anti-SS-A antibody titer of 114 (<110), but she was negative for other autoantibodies, such as antinuclear factor, anti-SS-B antibodies, rheumatoid factor, anti Jo-1 antibodies and anti-scl-70 antibodies.

An analysis of her serum revealed an IgG level of 1,700 mg/dL, IgA of 61 mg/dL, IgM of 41 mg/dL, IgE of 21 mg/dL, an albumin fraction level of 57 (>60) %, alpha-1 of 3.4%, alpha-2 of 9.2%, beta of 7.3 (>8.3) %, and gamma of 23.1 (<20) %, an angiotensin-converting enzyme (ACE) level of 25.0 (<25) IU/L, lysozyme of 16.7 (<10) μg/mL and a level of soluble interleukin-2 receptor (sIL-2R) of 3,570 (<519) U/mL. The patient was negative for the hepatitis B antigen and hepatitis C antibodies, and the titers of antibodies to Epstein-Barr virus and human T-cell leukemia virus type 1 were not elevated. An arterial blood gas analysis during room air breathing revealed a pH of 7.39, a PaCO_2_ of 37.0 Torr and a PaO_2_ of 75.3 Torr. Pulmonary function tests demonstrated a mild restrictive impairment [FVC of 2.09 L (79.8% predicted)], airway obstruction [FEV 1.0 of 1.56 L (67.8% predicted)] and a reduced percent diffusion capacity for carbon monoxide of 67.6%. A chest CT (shown in Figure 1B) and Ga-67 citrate scintigraphy showed no evidence of abnormal uptake. There was lymphocytosis (51%) with a CD4/CD8 ratio of 1.05 in her bronchoalveolar lavage fluid, but the transbronchial lung biopsy specimens did not lead to a definitive diagnosis. As there was little progression of her symptoms, she was discharged home with no treatment and was followed up for any changes.

**Figure 2:**
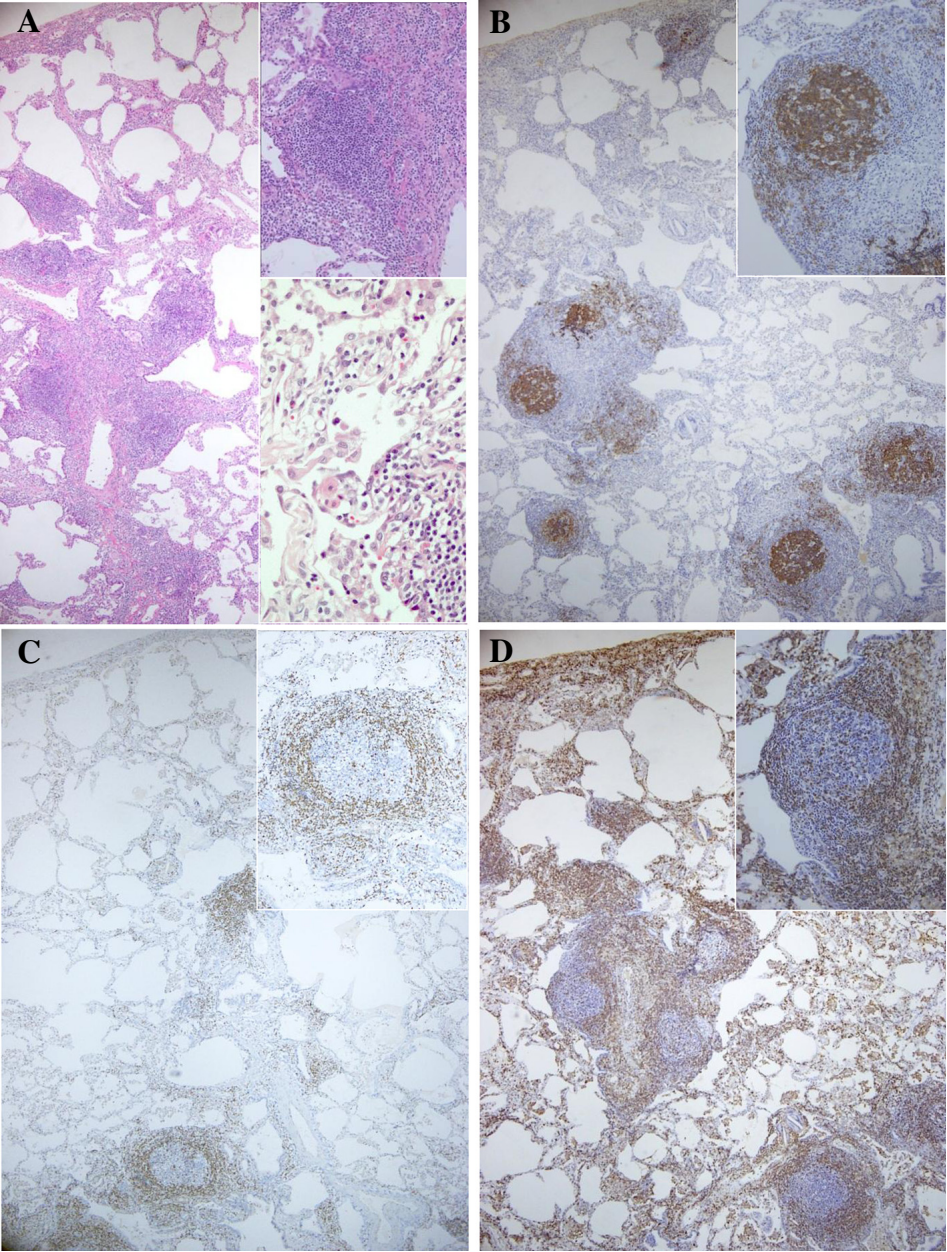
**A lung biopsy specimen of the left lower lobe, showing mild and homogeneous interstitial pneumonia, with the presence of numerous lymphoid follicles and no definite nuclear atypicality in the lymphocytes [hematoxylin-eosin stain; magnification ×40 (A), ×100 (insert upper), ×400 (insert lower): the boundary part between the lymphoid follicle (lower right) and surrounding tissue].** The immunohistochemical staining for L26, showing exclusively B-lymphocytes within the lymphoid follicles that exhibited a brownish reaction product [magnification ×40 **(B)**, ×100 (insert)]. The immunohistochemical staining for bcl-2, showing the distribution of bcl-2 positive lymphocytes around the centers of lymphoid follicles (reactive hyperplasia pattern) [magnification ×40 **(C)**, ×100 (insert)]. The immunohistochemical staining for UCHL-1, showing diffusely distributed T-lymphocytes in the alveolar walls or around the lymphoid follicles [magnification ×40 **(D)**, ×100 (insert)].

Five months later, she complained of the gradual development of xerostomia, exertional breathlessness with hypoxemia and a worsening of the chest shadows was observed, along with increases in the serum levels of sIL-2R (5,540 U/mL), lactate dehydrogenase (872 IU/L), ACE (32.9 IU/L) and lysozyme (27.5 μg/mL). The PaO_2_ was 64.8 Torr and the pulmonary function tests revealed a %FVC of 66.5% and a %FEV 1.0 of 63.6%. A positron emission CT scan showed an enhanced uptake, especially in the left lower lobe (S9), in which the early and delayed maximum standardized values (SUV max) were 2.9 and 3.4, respectively, but there was no enhancement in the extrapulmonary areas. The surgical lung biopsy specimens demonstrated an atypical pattern of cellular interstitial pneumonia (Figure 2A). Southern blot hybridization and the polymerase chain reaction were performed to assess the risk of malignant conditions. The infiltrating lymphocytes of the lung consisted of both B-cells and T-cells, however, the monoclonality of T-cells was proven by a Southern blot analysis for T-cell receptor gene rearrangement using the biopsied lung specimens. In terms of the immunoglobulin heavy chain gene rearrangement, neither Southern blot hybridization nor the polymerase chain reaction for complementarity determining region-3 showed any positive findings. Additionally, a histological examination of an inguinal lymph node, which was identifiable and could be biopsied in the extrapulmonary area, revealed no definitive evidence of lymphoma or other disease, and the results of the morphological analysis and gene testing of the bone marrow aspirate were regarded as negative**.** Moreover, the Schirmer and Rose Bengal dye tests were positive, and a lip biopsy, which revealed infiltration of two foci in 50 or more inflammatory cells within a 4 mm^2^ area of glandular tissue, confirmed a diagnosis of SS according to the American-European consensus criteria.

The patient was subsequently treated with methylprednisolone pulse therapy, followed by oral administration of prednisolone. The pulmonary lesion partly improved and has been kept in a stable condition with a maintenance dose of prednisolone alone (10 mg/day) for over three years.

## Discussion

In the present case, the history of dry eyes and dry mouth lasting more than three months, positive Schirmer and Rose Bengal dye tests, a positive salivary gland biopsy exhibiting infiltration of more than one focus score and the presence of autoantibodies to SS-A in the serum were observed. Furthermore, the patient had no history of radiation treatment, medical transplantation, anticholinergic drug use, and no evidence of HCV or HIV infection, and her clinical and laboratory examinations did not lead to a diagnosis of sarcoidosis or lymphoma. Based on these findings, a diagnosis of SS was confirmed according to the American-European consensus criteria [4].

SS is characterized by the lymphocytic infiltration of the exocrine glands, together with a polyclonal B-cell activation. Pulmonary involvement is common in SS. Constantopoulos *et al*. [5] classified the lung diseases in patients with SS into interstitial pulmonary disease, small airway disease, desiccation of the upper respiratory tract, large airways obstruction and lymphoproliferative disorders. It has been reported that malignant tumors are common in patients with lymphoproliferative disorders, and that the coexistence rate of SS with lymphoma is approximately 5% [6]. Lymphoma of the lung is substantially less common than lymphoma at other sites in SS. However, Kassan *et al.*[7] reported that the risk of malignant lymphoma in SS patients was 44 times higher than that in healthy people. Lymphoproliferative disorders are thought to arise from lymphoepithelial lesions in which there are close interactions among epithelial cells, T-cells and B-cells. However, in most cases of lymphoma/SS, the malignant transformation from a benign lymphoproliferative process to a malignancy is believed to result from chronic excessive B-cell stimulation [8].

In the present case, the histopathological findings included a diffuse inflammatory infiltrate that comprised mainly lymphocytes, plasma cells and histiocytes in the parenchymal interstitium and loose-binding connective tissue (Figure 2A). Immunohistochemically, B-cells constituted lymphoid follicles at the centers, and a hyperplastic pattern of distribution was observed by bcl-2 staining (Figure 2B and C). On the other hand, the infiltrative cells were mainly T-cell lymphocytes (Figure 2D). In addition, the clonality of T-cells, but not of B-cells, was proven by the gene rearrangement analyses. However, as no definite nuclear atypicality in the lymphocytes was observed (Figure 2A); it was difficult to morphologically classify the present case under the WHO classification of lymphomas and to clarify whether it was a case of primary pulmonary lymphoma or a lymphoproliferative disorder complicated with SS. Therefore, we made a diagnosis of diffuse pulmonary lymphoproliferative disorder coincident with the monoclonal proliferation of T-lymphocytes.

In diffuse pulmonary diseases associated with SS, a non-specific interstitial pneumonia (NSIP) pattern, an organizing pneumonia (OP) pattern, a usual interstitial pneumonia (UIP) pattern and LIP are common. Extranodal marginal zone lymphoma of mucosa-associated lymphoid tissue (MALT lymphoma) is a representative example of a neoplastic pulmonary disorder that develops in patients with SS [9]. It is well known that SS is associated with lymphoid interstitial pneumonia. Travis *et al.*[10] reported that the rate of coexistence of LIP in SS was approximately 1%, and that 9 to 25% of LIP cases were associated with SS. In case of LIP, the infiltrates are characterized by dense, diffuse infiltration predominantly in the alveolar septae, including both polyclonal lymphocytes and plasma cells, whereas other lymphomas produce monoclonal infiltrates. As the infiltrates had spread predominantly into loose-binding connective tissue in our case, the diagnosis of a NSIP pattern with diffuse lymphoid hyperplasia associated with SS might be more adequate than LIP. However, as they exhibited TCR monoclonality, T-cell neoplasms needed to be taken into consideration.

Primary pulmonary lymphomas (PPL) represent less than 1% of primary malignant lung tumors, 1% of lymphomas and only 3-4% of extranodal lymphomas. Most of the PPLs are MALT lymphomas, which usually have an indolent course [9]. Currently, no standard therapy for non B-cell lymphoma, especially of the lung, has been established due to its rarity and poor prognosis due to its aggressive nature in many cases [11]. In most of the previous reports, although immunohistochemistry or flow cytometry studies were used to define the lineage of the cells, genotypic assessments were not necessarily reported. These histopathological examinations usually revealed cytological dysplasia, homogenous populations of cells and architectural effacement. Nevertheless, a rare case was reported in which the diagnosis of T-cell lymphoma was made mainly depending on a TCR gene rearrangement [12]. In the present case, the relatively slow progression, lack of cytological atypicality and the stabilization of the disease condition by treatment with systemic corticosteroid alone were irreconcilable with the previously-reported cases of primary pulmonary T-cell lymphoma. Furthermore, even though monoclonality is thought to be a characteristic of neoplasia, it does not always define malignancy. For example, it is not present in drug-induced pseudolymphoma syndrome, which is usually improved by the administration of systemic steroids after cessation of exposure to the causative agents [13].

## Conclusions

In the present case, although monoclonality of the infiltrating T-cells was proven, the clinical course and the examination findings were inconsistent with the previously-reported cases of primary pulmonary T-cell lymphoma. It is currently unclear whether this case has malignant potential, but the patient will be closely followed up for potential malignancy. The diagnosis of malignant lymphoma or lymphoproliferative diseases should be made in a comprehensive manner clinically, pathologically and cytogenetically.

## Consent

Written informed consent was obtained from the patient for publication of this case report and accompanying images. A copy of the written consent is available for review by the Editor-in-Chief of this journal.

## Competing interests

The authors declare that they have no competing interests.

## Authors’ contributions

HO, CY and HI drafted and revised the manuscript. KK, HK and JK were involved in the patient diagnosis and management. All authors read and approved the final manuscript.
